# Probing the origins of metazoan formin diversity: Evidence for evolutionary relationships between metazoan and non-metazoan formin subtypes

**DOI:** 10.1371/journal.pone.0186081

**Published:** 2017-10-05

**Authors:** David Pruyne

**Affiliations:** Department of Cell and Developmental Biology, State University of New York Upstate Medical University, Syracuse, NY, United States of America; Vanderbilt University, UNITED STATES

## Abstract

Formins are proteins that assist in regulating cytoskeletal organization through interactions with actin filaments and microtubules. Metazoans encode nine distinct formin subtypes based on sequence similarity, potentially allowing for great functional diversity for these proteins. Through the evolution of the eukaryotes, formins are believed to have repeatedly undergone rounds of gene duplications, followed by diversification and domain shuffling, but previous phylogenetic analyses have shed only a little light on the specific origins of different formin subtypes. To improve our understanding of this in the case of the metazoan formins, phylogenetic comparisons were made here of a broad range of metazoan and non-metazoan formin sequences. This analysis suggests a model in which eight of the nine metazoan formin subtypes arose from two ancestral proteins that were present in an ancient unikont ancestor. Additionally, evidence is shown suggesting the common ancestor of unikonts and bikonts was likely to have encoded at least two formins, a canonical Drf-type protein and a formin bearing a PTEN-like domain.

## Introduction

The formin family was first recognized when it was noted that proteins from several animals and budding yeast share two regions of homology with the "Formin" product of the mouse *limb deformity* locus: a proline-rich formin homology-1 (FH1) domain and a unique formin homology-2 (FH2) domain [1]. Further analysis has shown formins are nearly ubiquitous among eukaryotes, including organisms as diverse as ciliates, green plants, and amoebas. In many of these organisms, formins are known to promote the organization of substructures of the actin cytoskeleton (reviewed in [2–4]). *In vitro* studies have shown that homodimers of the FH2 domain of many formins interact directly with actin, often promoting the nucleation of new actin filaments and influencing the rate and extent of elongation at the filament barbed end (reviewed in [5,6]). The FH1 domain directly binds the actin monomer-binding protein profilin, and in conjunction with profilin, can accelerate the elongation of formin-bound actin filaments [7,8]. In addition to affecting actin dynamics, many formins bind microtubules and microtubule-binding proteins through FH2 domains and other motifs, and many formins have been shown to promote microtubule stability *in vivo* [2].

A detailed analysis of conserved motifs in diverse FH2 domains had suggested that the FH2 fold is likely to have arisen only once during the evolution of eukaryotes [9]. Among various formins, the FH2 domain has been coupled to a variety of different other structural domains, suggesting this family has been subject to patterns of gene duplications followed by divergence and domain shuffling. However, tracing the evolutionary history of the formins from presumptive common ancestors has been difficult, due in large part to weak constraints on all but a few FH2 domain amino acid residues [9]. Previous phylogenetic analyses have been largely limited to defining conserved subtypes of formins within different groups of organisms, without inferring much about relationships between those subtypes [9–12]. However, since those earlier studies, the sequencing and annotation of additional genomes from a broad array of metazoan and non-metazoan organisms has provided additional data points that help to begin filling in our picture of the evolution of the formin family.

**Fig 1:**
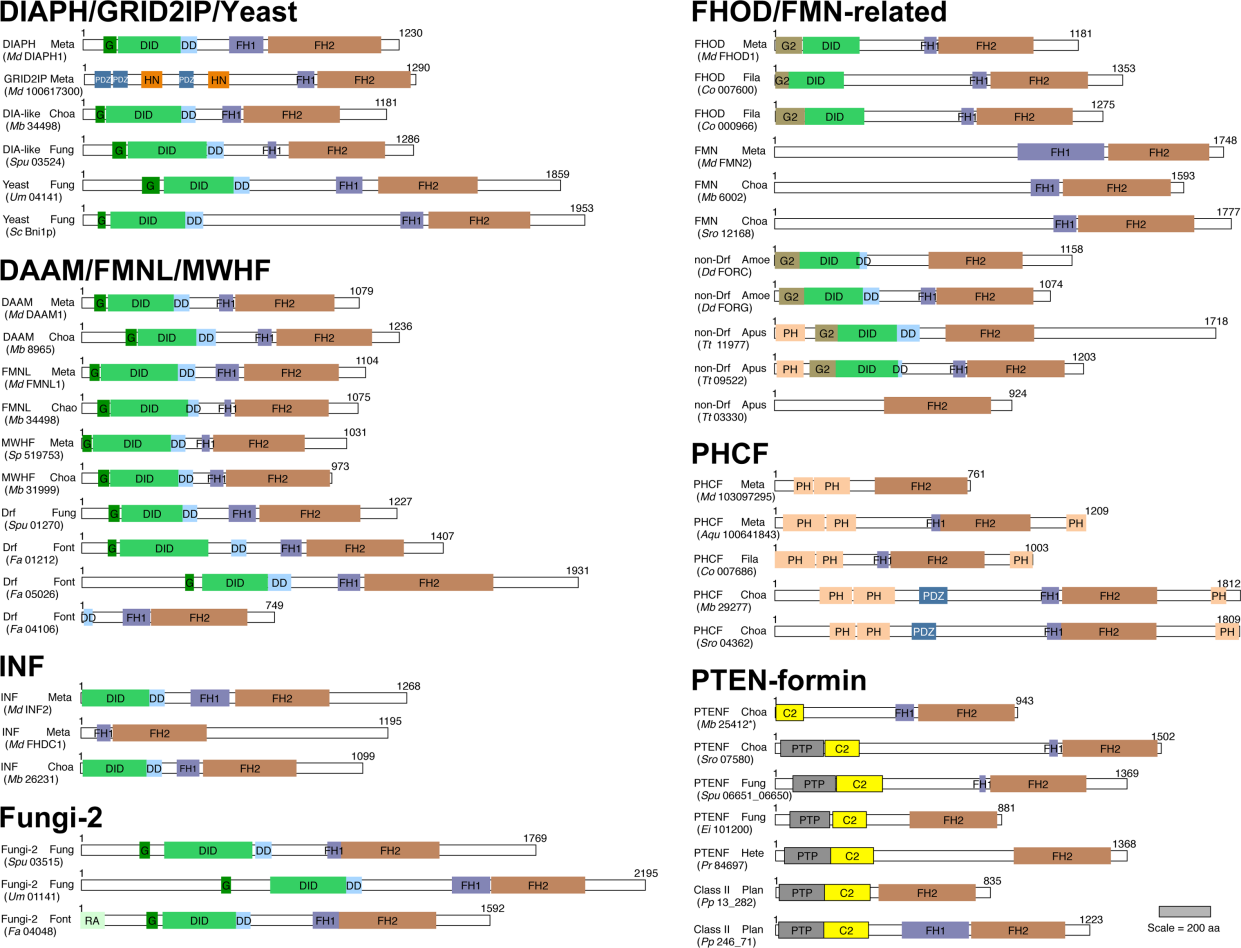
Domain organizations of metazoan and non-metazoan formins. Predicted domain organizations are shown for representative proteins from seven groups of formins identified based on similarity of FH2 domain or (for PTEN-formins) PTEN-like domain sequences. Indicated for each protein is the formin subtype and the superphylum/kingdom of the source organism, with parenthetical annotation of specific species and protein identity, as per S1 Table. Domains shown to scale are: GTPase-binding (G, dark green), alternative GTPase-binding (G2, olive), DID (medium green), DD (light blue), PDZ (dark blue), HN (orange), FH1 (purple), FH2 (red), PH (tan), RA (light green), PTP (gray), and C2 (yellow). Represented are the superphyla/kingdoms metazoa (Meta), choanoflagellata (Choa), filasterea (Fila), fungi (Fung), fonticulida (Font), apusozoa (Apus), amoebozoa (Amoe), plantae (Plan), and heterokonta (Hete), and the species *M*. *domestica* (*Md*), *M*. *brevicollis* (*Mb*), *S*. *punctatus* (*Spu*), *U*. *maydis* (*Um*), *S*. *cerevisiase* (*Sc*), *S*. *purpuratus* (*Sp*), *F*. *alba* (*Fa*), *C*. *owczarzaki* (*Co*), *S*. *rosetta* (*Sro*), *D*. *dictyostelium* (*Dd*), *T*. *trahens* (*Tt*), *E*. *intestinalis* (*Ei*), *P*. *ramorum* (*Pr*), or *P*. *patens* (*Pp*). The scale bar indicates distances in amino acids.

The animal (metazoan) formins provide a group whose evolutionary origins are particularly interesting. Metazoan formins can be categorized into nine subtypes based on the degree of similarity of their FH2 domain sequences [13], designated here: disheveled-associated activator of morphogenesis (DAAM) proteins, diaphanous (DIAPH) proteins, formin homology domain containing (FHOD) proteins, canonical formins (FMN), formin-like (FMNL) proteins, glutamate receptor ionotropic delta 2-interacting proteins/delphilins (GRID2IP), inverted formins (INF), multiple wing hairs-related formins (MWHF), and pleckstrin homology (PH) domain-containing formins (PHCF). Typical members of all these subtypes possess the conserved FH1-FH2 domain module, but otherwise vary in their domain organization (Fig 1).

The most common domain organization found among metazoan formins is that of the diaphanous-related formin (Drf), as found in DIAPH, DAAM and FMNL formins. Drfs have an extended N-terminus that includes a Rho-family GTPase-binding domain (designated here as, G) followed by an armadillo repeat-rich diaphanous inhibitory domain (DID) and an α-helical dimerization domain (DD) [14,15]. For typical Drfs, a C-terminus extending beyond the FH2 domain encodes one or two motifs that show varying degrees of similarity to Wiskott Aldrich Syndrome Protein Homology-2 (WH2) motifs [16–18]. Such motifs sometimes assist in filament nucleation or barbed end binding, and sometimes promote additional activities such as severing or bundling of actin filaments (reviewed in [19,20]). For many Drfs, some of these motifs also have the ability to bind to the DID to establish an autoinhibited conformation, and in such cases, the motif is also referred to as a diaphanous autoregulatory domain (DAD) [21,22]. This autoinhibitory DID/DAD interaction can be weakened by the binding of a RhoGTPase to the G and the DID, providing a partial explanation for the observation that many formins are regulated *in vivo* by RhoGTPases [14,15,23–25].

Several additional metazoan formin subtypes resemble Drfs with modest alterations (Fig 1). MWHFs lack obvious C-terminal DAD/WH2-like motifs, while INFs resemble N-terminally truncated Drfs, with some isoforms lacking a G domain, and others also lacking DID and DD sequences [10,13]. FHODs lack a recognizable DD, and they encode an alternative RhoGTPase-binding domain (termed here, G2) that adopts a fold that is distinct from the Drf-type G domain [26]. The remaining metazoan formin subtypes differ from Drfs more drastically. The N-termini of FMNs are generally predicted to be helical and hydrophilic with no defined fold, and their C-termini lack recognizable WH2-like motifs. The N-termini of GRID2IPs encode one to three sets of postsynaptic density protein 95/ Drosophila disc large tumor suppressor 1/zonula occludens-1 protein (PDZ) domains and Harmonin N-terminus-like (HN) domains, while their C-termini also lack identifiable motifs [27–29]. Finally, PHCFs typically have a pair of N-terminal PH domains and/or a single C-terminal PH domain, but no detectable G, DID, DD, or WH2-like sequences [13].

We have relatively little insight as to how this diversity of metazoan formins arose. Members of all nine subtypes are widespread across many metazoan phyla. It may be particularly telling that the sponge *Amphimidon queenslandica*, a member of the basal metazoan phylum Porifera, encodes formins from all nine subtypes. This suggests the nine subtypes were already in existence in the last common ancestor of the metazoans [13]. Comparisons of metazoan FH2 domains with those from the choanoflagellate *Monosiga brevicollis*, a close relative of metazoans, have suggested at least four metazoan subtypes (DAAM, FMNL, FMN, INF) were already present in the common metazoan/choanoflagellate ancestor [9,12]. Comparisons to the formins of yeasts and other fungi have shown a general conservation of the Drf-type domain organization, but provided little additional insight into specific relationships with metazoan subtypes. However, comparisons to FH2 sequences of the distantly related slime mold *Dictyostelium discoideum* revealed a clear kinship between metazoan FMNs and several *D*. *discoideum* formins [9,11,12].

Using a large set of sequenced non-metazoan genomes, this analysis attempts to revisit and expand on those earlier studies. Revealed are previously unappreciated relationships between specific metazoan formin subtypes and the formins of fungi, amoebozoans, and other non-metazoans. The results support a model in which eight of the nine metazoan formin subtypes originated from two formins that were present in an ancestral unikont.

## Materials and methods

### Identification of FH2 domain-containing proteins

Metazoan formin sequences considered here were identified previously [13]. Unikont formins were identified by the same methods: through accessing protein and translated nucleotide databases via the website of the National Center for Biotechnology Information (NCBI) (https://www.ncbi.nlm.nih.gov/) and the website of Ensembl Genomes (www.ensemblgenomes.org; [30]), and searching these with the Basic Local Alignment Search Tool [31]. For unikont organisms, to help ensure that all FH2 domains were identified for a given species, each species dataset was subjected to search queries based on the FH2 domains from *Mus musculus* DAAM1, DIAPH1, FMN2, GRID2IP, FHDC1 (an INF protein), INF2, FMNL1, and FHOD1, *Strongylocentrotus purpuratus* LOC100890634 (a PHCF protein), and *Caenorhabditis elegans* FOZI-1 (a highly divergent FMNL-related protein). Non-exhaustive searches for formins in bikont species were performed by searching the relevant species dataset on the Ensembl Genomes website using the search term, "FH2". All identified formins are listed by species in S1 Table. In cases where two adjacent genes appeared to encode pieces of the same formin, both genes are used to identify the formin in S1 Table and in all phylogenetic trees.

### Domain analysis

Similar to as done previously for metazoan formins [13], the boundaries of FH2 domains in non-metazoan formin sequences were predicted by Conserved Domain Search [32] against the NCBI Conserved Domain Database superset [33], while most other structural domains were identified by the Protein HomologY Recognition Engine 2 (PHYRE^2^) (www.sbg.bio.ic.ac.uk/phyre2/html/page.cgi?id=index; [34]). Predictions accompanied by a confidence of homology ≥ 95% were considered to be likely. FH1 domains were manually identified as all segments of two or more adjacent prolines plus all intervening sequences, located N-terminal to the FH2 domain. Due to the very loose consensus for DAD and WH2-like motifs and the difficulty in their identification, these were not considered in this analysis.

### Multiple sequence alignments and estimation of phylogenies

Amino acid sequence alignments were performed in MegAlign (ver 13.0.0) of the Lasergene software suite (DNASTAR, Madison, WI) using Clustal W [35], with the default settings (Gap penalty 10, gap length penalty 0.2) and a Gonnet Series matrix. Gross misalignments, typically due to large gaps or long insertions in individual sequences, were corrected manually. Sequence alignments are presented in the file S1 Text.

Evolutionary histories were inferred from the sequence alignments by applying the Maximum Likelihood (ML) method using the LG model [36] + G (using a discrete Gamma distribution with 5 categories to model evolutionary rate differences among sites; [37]) in the MEGA6 program [38]. The LG + G model was selected from 48 models after producing the lowest Bayesian Information Criterion score for each sequence alignment [39]. For alignments composed of complete or nearly complete sequences only, analysis by the MEGA6 program was set to exclude positions in the sequences alignments that were only partially occupied. In sequences alignments in which some positions were unoccupied in only a small minority of sequences due to incomplete sequence information or truncation of a protein, the exclusion threshold in MEGA6 was set at below 90% or 95% occupancy. Unrooted phylogenetic trees were generated in MEGA6.

## Results and discussion

**Fig 2:**
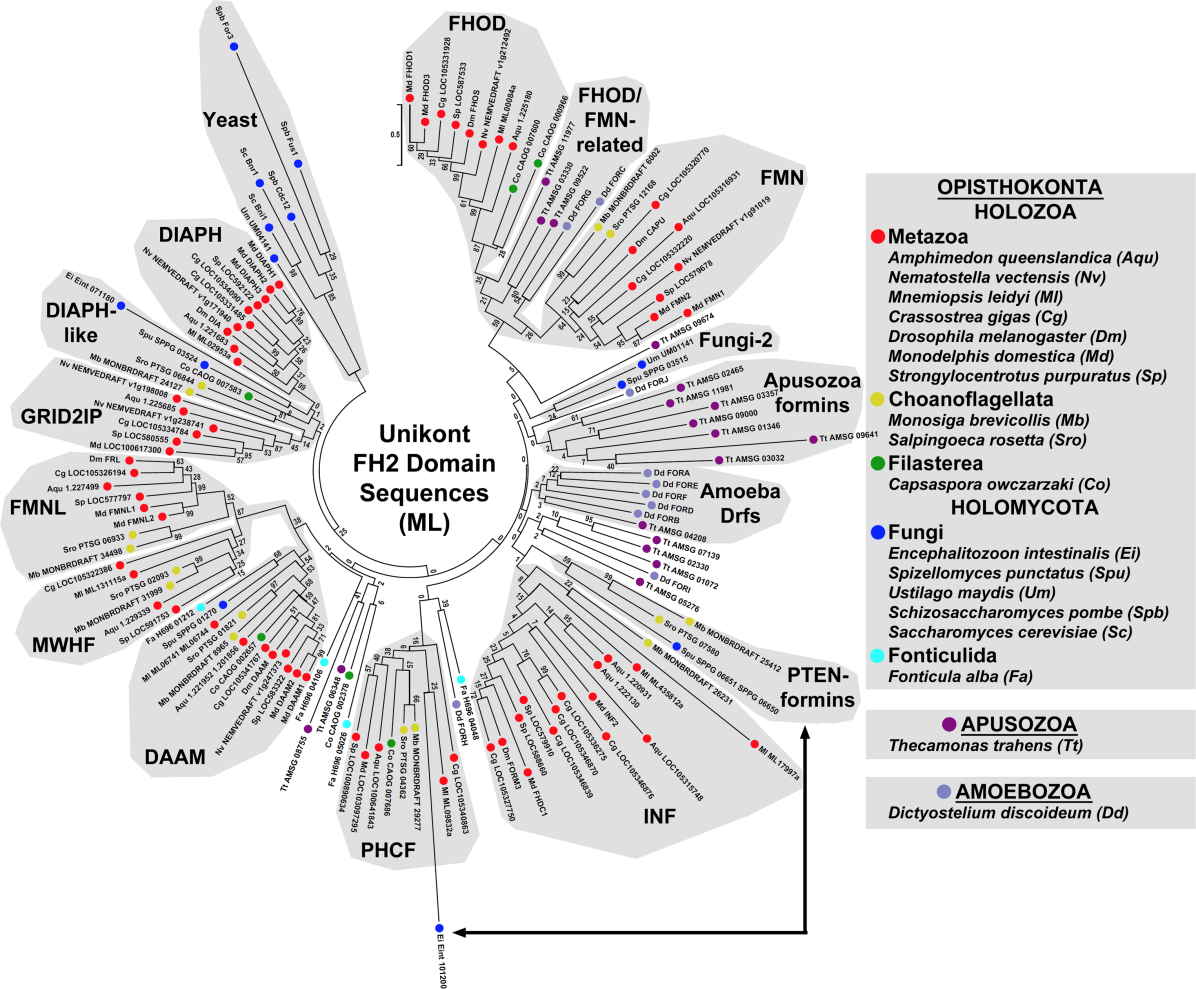
Unrooted ML phylogenetic tree of metazoan and non-metazoan FH2 domain sequences. The evolutionary history for 136 FH2 domain amino acid sequences from the indicated unikont species was inferred by the ML method using the LG + G model for 251 fully occupied positions. Observed groups of formins based on inferred FH2 sequence relatedness include the nine metazoan formin subtypes (DAAM, DIAPH, FHOD, FMN, FMNL, FMNLR, GRID2IP, INF, PHCF), two subtypes enriched for fungal formins (Yeast, Fungi-2), a novel group of opisthokont formins with N-terminal PTEN-like domains (PTEN-formins), and looser groups of amoebozoan Drf-type formins (Amoeba Drfs) and apusozoan formins (Apusozoa formins). Other formins were ungrouped or associated with multiple subtypes to similar degrees. The latter category included opisthokont formins related to GRID2IP and DIAPH subtypes (DIAPH-like formins), and apusozoan and amoebozoan formins related to FHOD and FMN proteins (FHOD/FMN-related formins). The formin Eint_101200 of the microsporidian fungus *E*. *intestinalis* is depicted within the metazoan PHCF subtype, but this may be an artifact based on the absence of any PH domain and the presence of an N-terminal PTEN-like domain in this formin. Its probable relationship to the PTEN-formins is indicated by a double-arrow. All bootstrap values are indicated, and the scale bar indicates the number of substitutions per site for branch lengths.

**Fig 3:**
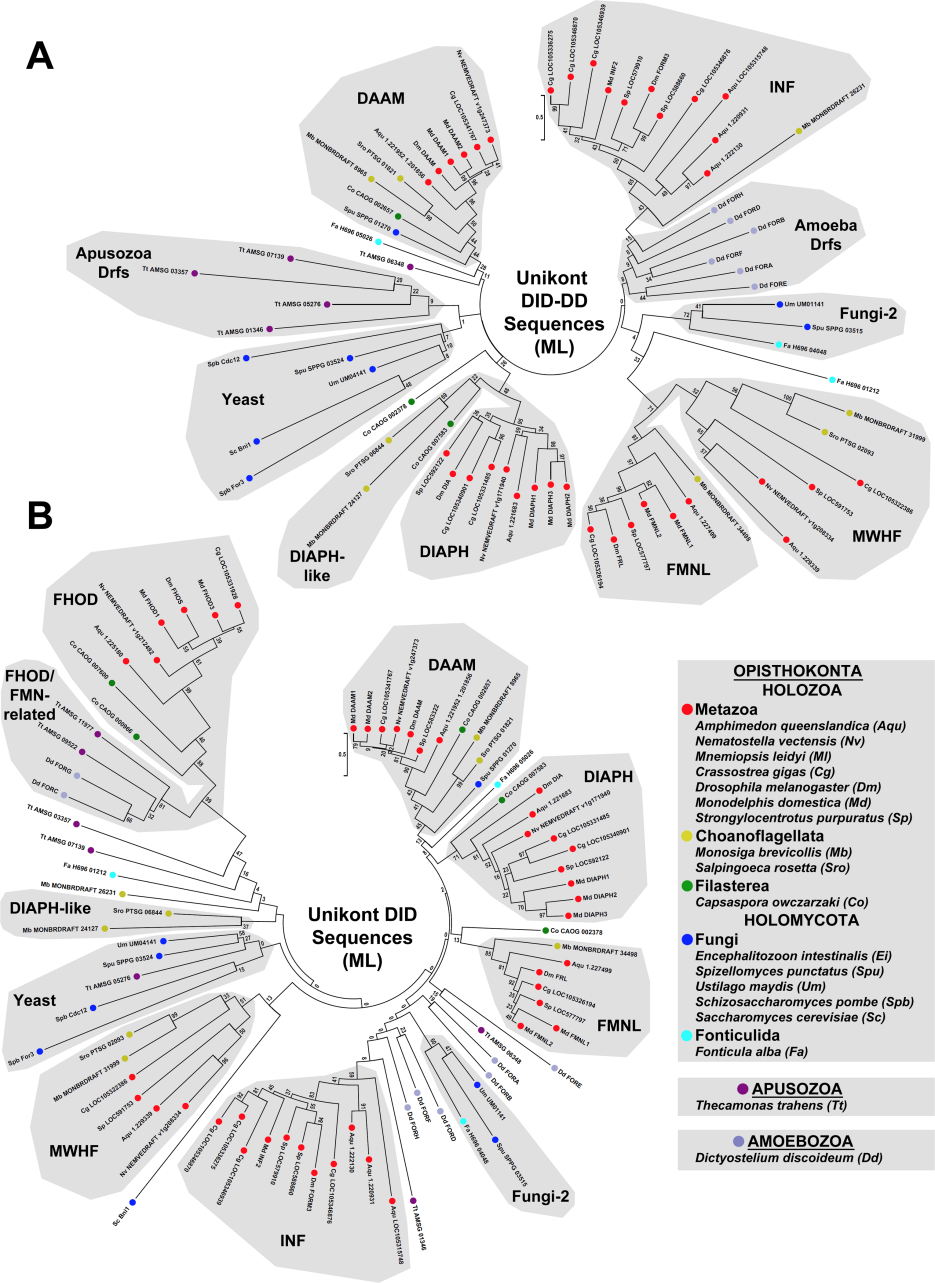
Unrooted ML phylogenetic trees of N-terminal domains from metazoan and non-metazoan formins. Evolutionary histories for (A) 69 DID-DD sequences and for (B) 82 DID sequences of the indicated unikont species were inferred by the ML method using the LG + G model for 266 positions that were occupied in ≥ 90% of DID-DD sequences, or 172 positions that were occupied in ≥ 95% of DID sequences. The formin groups shown here generally recapitulate those based on FH2 domain phylogeny (Fig 2), with some exceptions, particularly when the shorter DID sequence is considered. All bootstrap values are indicated, and the scale bars indicate the number of substitutions per site for branch lengths.

**Fig 4:**
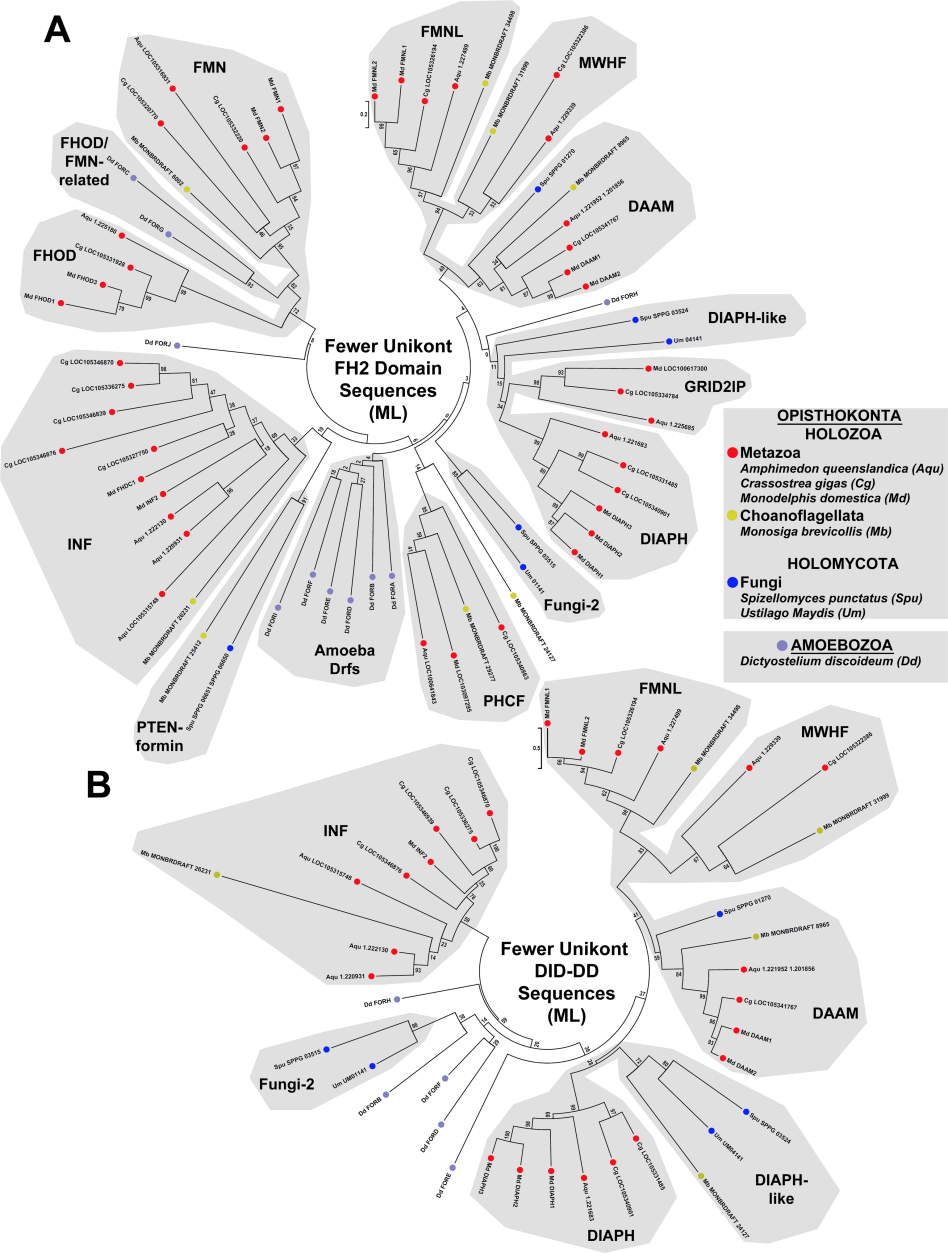
Unrooted ML phylogenetic trees estimated using fewer formin sequences highlight relationships between fungal and metazoan formins. To help clarify some ambiguities of connectivity in dense regions of phylogenetic trees shown in Figs 2 and 3, a smaller number of (A) FH2 domain and (B) DID-DD sequences were considered separately. Thus, evolutionary histories for 65 FH2 domain and 39 DID-DD sequences from the indicated unikont species were inferred by the ML method using the LG + G model for 314 positions that were fully occupied in all FH2 domain sequences, or for 244 positions that were occupied in ≥ 90% of DID-DD sequences. A notable change is in the placement of the formins UM_04141 of the basidiomycote fungus *U*. *maydis* and SPPG_03524 of the chytrid fungus *S*. *punctatus*. These formins clustered with the Yeast subtype in some or all trees of Figs 2 and 3, but area associated with the DIAPH-like formin MONBRDRAFT_24127 of the choanoflagellate *M*. *brevicollis* in the simpler trees of (A) and (B). All bootstrap values are indicated, and the scale bars indicate the number of substitutions per site for branch lengths.

The FH2 domain is thought to have arisen only once during evolution [9], suggesting that the diversity of metazoan formin subtypes has resulted from repeated duplications of ancestral formins followed by divergence of sequence. To probe for evidence of such duplication/divergence events, FH2 domain sequences for formins belonging to seven metazoan species (sponge *A*. *queenslandica*, ctenophore *Mnemiopsis leidyi*, sea anemone *Nematostella vectensis*, oyster *Crassostrea gigas*, fruit fly *Drosophila melanogaster*, opossum *Monodelphis domestica*, and sea urchin *Strongylocentrotus purpuratus*) were aligned with those from eleven non-metazoan species (see below), and a ML tree was estimated (Fig 2). When DID, or DID and DD (DID-DD) domains were present in these formins, their sequences were also aligned and ML trees estimated (Fig 3). For some regions of these trees, the high density of branches resulted in poorly supported nodes with low bootstrap values. In an attempt to produce simpler trees whose nodes might be resolved with more robust support, phylogenies were also estimated for smaller numbers of aligned FH2 domains and DID-DD sequences (Fig 4).

In all these trees, metazoan formins clustered into nine groups that recapitulated the metazoan subtypes previously identified [13]. Three general classes of relationships could be observed between the metazoan formin subtypes and the non-metazoan formins. In the first class, non-metazoan formins appeared to be members of a metazoan subtype. This was interpreted to suggest that the origin of this formin subtype predated the divergence of the lineages of the metazoans and that particular non-metazoan. In the second class, non-metazoan formins appeared to be similarly related to two or more metazoan subtypes. This was interpreted to suggest that those metazoan subtypes derived from a common ancestral formin present in the last shared ancestor of the metazoans and that particular non-metazoan. Finally, in the third class there was no apparent particular relatedness between a non-metazoan formin and any metazoan subtype, which was interpreted to reflect either a real absence of shared ancestry, or an obscuring of any relationship by extensive sequence divergence. Below, relationships are examined with formins of organisms of increasing divergence, in an attempt to trace the probable course of metazoan formin diversification backward through evolution.

### Holozoan formins and evidence for a recent origin of GRID2IP formins

Metazoa belongs to the larger group of organisms, Holozoa, which also contains the very closely related Choanoflagellata, and the somewhat more distant Filasterea [40,41]. Thus, to consider relationships among formins of the closest relatives to metazoans, twenty-two formins were identified and examined from two choanoflagellates, *Salpingoeca rosetta* and *M*. *brevicollis*, and from one filasterean, *Capsaspora owczarzaki* (S1 Table). Of these, sixteen formins appeared to be a member of a metazoan subtype based on estimated phylogeny of FH2 domains sequence (Fig 2). Specifically, all three non-metazoan holozoans encode apparent DAAM and PHCF proteins, each choanoflagellate encodes one apparent representative each of the FMNL, MWHF, INF, and FMN subtypes, and the filasterean encodes two apparent FHOD subtype formins. Supporting these FH2-based similarities, each putative homolog was predicted to have domain organization conserved with its metazoan counterpart: G-DID-DD-FH1-FH2 for the DAAMs, FMNLs, and MWHFs; DID-DD-FH1-FH2 for the INFs; G2-DID-FH1-FH2 for the FHODs; X-FH1-FH2 for the FMNs (where X represents extended sequence for which PHYRE^2^ predicted no known domains); and PH-PH-FH1-FH2-PH for the PHCFs (Fig 1). Only the choanoflagellate PHCFs deviated from this by encoding a PDZ domain N-terminal to their FH1 domain (PH-PH-PDZ-FH1-FH2-PH), but absence of this PDZ domain from filasterean and metazoan PHCFs suggests it is a choanoflagellate-specific innovation. Also supporting these subtype assignments, when DID-DD or DID sequences were present and compared, these sixteen holozoan formins again clustered with the appropriate metazoan subtype (Fig 3).

**Fig 5:**
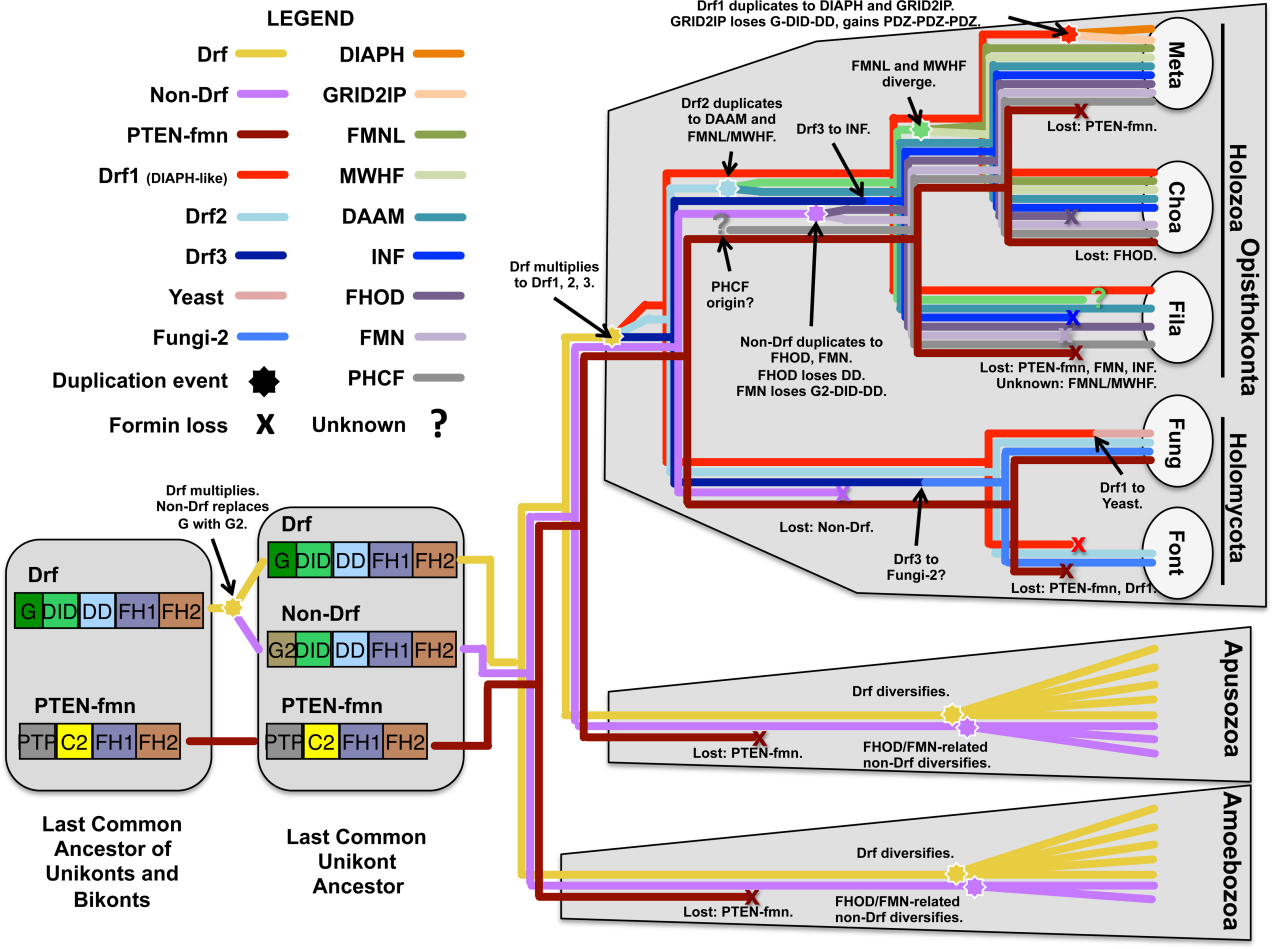
Formin diversification and loss in the unikonts. Presented here is a model for the evolution of the formins from a common unikont/bikont ancestor on the left, to modern taxa on the right, including Amoebozoa, Apusozoa, and members of Opisthokonta, including the holozoan groups Metazoa (Meta), Choanoflagellata (Choa), and Filasterea (Fila), and the holomycote groups Fungi (Fung), and Fonticulida (Font). Colored traces indicate inheritance of indicated formin isoforms, while duplication or multiplication events are shown with stars, loss of isoforms is indicated by "x", and unknown events are indicated by "?". The positions of events along branches are not meant to imply relative timing of events, but are for visual clarity. Based on the presence of similar formins in unikont and bikont organisms, the last common ancestor shared by unikonts and bikonts is likely to have encoded a Drf with G-DID-DD-FH1-FH2 domain organization (yellow), and a PTEN-formin with PTP-C2-FH1-FH2 domain organization (dark red). PTEN-formins may have been lost from amoebozoan and apusozoan lineages, and from many opisthokont lineages, but were retained in choanoflagellates and some fungi. In the lineage leading to the unikonts, the Drf is likely to have duplicated, with the replacement of the Drf-type G domain in one formin with a structurally dissimilar G2 domain, to produce a non-Drf with G2-DID-DD-FH1-FH2 domain organization (purple). In the opisthokonts, the non-Drf was lost from the holomycote lineage, but duplicated in the holozoan lineage, with one isoform losing its DD to become the G2-DID-FH1-FH2 FHOD subtype (dark purple), and another losing G2-DID-DD to become the X-FH1-FH2 FMN subtype (light purple). In the apusozoan and amoebozoan lineages, the G2-DID-DD-FH1-FH2 domain organization was retained in their FHOD/FMN-related non-Drfs. The G-DID-DD-FH1-FH2 Drfs appear to have diversified independently in the amoebozoan, apusozoan, and opithokont lineages. In the opisthokont lineage, the ancestral Drf is likely to have multiplied into at least three Drfs before the division of holozoa and holomycota. One resultant Drf (Drf1, red) was DIAPH-like. Among the fungi, this DIAPH-like formin gave rise to the Yeast subtype (pink), while in the metazoans lineage, shortly after its divergence from the choanoflagellates, DIAPH-like Drf1 duplicated, with one formin becoming the conserved G-DID-DD-FH1-FH2 DIAPH subtype (dark orange), and the other replacing its N-terminal G-DID-DD to become the PDZ-PDZ-HN-PDZ-HN-FH1-FH2 GRID2IP subtype (light orange). A second ancestral opisthokont Drf (Drf2, light blue) diversified in the holozoan lineage to give rise to the FMNL (green), MWHF (light green), and DAAM (dark teal) subtypes, while producing in the holomycote lineage fungal and fonticulid formins related to the FMNL/MWHF/DAAM subtypes. At least one additional Drf (Drf3, indigo) must have been present in the ancestral opisthokont to account for the holomycote G-DID-DD-FH1-FH2 Fungi-2 subtype (medium blue), and possibly the holozoan DID-DD-FH1-FH2 INF subtype (dark blue). The PHCF subtype (gray), with domain organization PH-PH-FH1-FH2-PH, is widespread among holozoans, but neither domain organization nor FH2 sequences provides any clues to its origin.

Each choanoflagellate and the filasterean also encode a Drf-type G-DID-DD-FH1-FH2 formin whose DID-DD sequence clustered with metazoan DIAPHs with moderate support (bootstrap value 48, Fig 3A). However, based on FH2 domain sequence, these formins were not unambiguously members of the DIAPH subtype. Instead, their branches on the FH2 domain tree were associated with a node that joined metazoan DIAPH and GRID2IP subtypes as a larger super-group (Fig 2). In the simpler FH2 domain tree (Fig 4A), this entire DIAPH/DIAPH-like/GRID2IP super group had modest support with bootstrap value 20 for the connecting node, and a more strong support (bootstrap value 39) for the node connecting the metazoan and choanoflagellate formins, only. The distinct PDZ-HN-rich N-termini of metazoan GRID2IPs (Fig 1) resemble other non-formin proteins, such as animal and choanoflagellate whirlin homologs, but are not similar to any non-metazoan formin, suggesting they are a metazoan innovation. A likely explanation for these relationships is that an ancestral holozoan DIAPH-like formin duplicated in the metazoan lineage shortly after its divergence from the choanoflagellates (Fig 5). One of the resulting formins retained its ancestral G-DID-DD-FH1-FH2 domain organization and become the metazoan DIAPH, while the N-terminus of the other was replaced to produce the metazoan GRID2IP with PDZ-PDZ-HN-PDZ-HN-FH1-FH2 domain organization (Fig 5).

The choanoflagellates and filasterean also each encode one orphan formin that did not group with any particular group or groups of metazoan formins. For the Drf-type filasterean orphan, CAOG_02378, it is unclear if its lack of relatedness reflects incomplete or incorrect sequence data, poor conservation of sequence, or derivation from an unrelated formin subtype in the filasterean lineage. The choanoflagellate orphan formins (PTSG_07580 and MONBRDRAFT_25412) formed a well-supported group (bootstrap value 99) based on their FH2 domain sequences (Fig 2). PHYRE^2^ predicted the N-terminus of the *S*. *rosetta* orphan encodes a phosphatase and tensin (PTEN)-like domain, which is a composite domain that includes a protein tyrosine phosphatase (PTP)-like fold and a membrane-binding C2-like fold (Fig 1). The *M*. *brevicollis* orphan was predicted to contain a C2-like fold, only (Fig 1). Based on these features, these choanoflagellate formins are referred to here as PTEN-formins.

Summarizing these observations for the formins of Holozoa, distinct FMNL, MWHF, FMN, and INF proteins were present at least before the divergence of choanoflagellates and metazoans, while DAAM, PHCF, FHOD, and DIAPH-like proteins are likely to have existed in the common holozoan ancestor of filastereans, choanoflagellates, and metazoans (Fig 5). Distinct DIAPH and GRID2IP proteins very likely arose by duplication of the ancestral DIAPH-like formin very early in the metazoan lineage, after its divergence from the choanoflagellates (Fig 5).

### Holomycota formins and evidence for conserved Drf-related subtypes among fungi

To consider relationships among formins of organisms somewhat more diverged from metazoans, proteins were examined from members of Holomycota, a sister group to Holozoa within the larger group Opisthokonta [42,43]. Specifically, seventeen formins were examined from one representative of Fonticulida, *Fonticula alba*, and from five representatives of four phyla from Fungi, *Saccharomyces cerevisiae* and *Schizosaccharomyces pombe* of Ascomycota, *Ustiago maydis* of Basidoimycota, *Spizellomyces punctatus* of Chytridiomycota, and *Encephalitozoon intestinalis* of Microsporidia (S1 Table).

Consistent with previous analyses [9–12], many fungal formins fell into two groups composed mostly of Drf-type formins (Fig 1). One of these groups included all the budding and fission yeast formins plus additional fungal formins, and is called here the Yeast subtype, while the second group is called here the Fungi-2 subtype (Figs 2 and 3). Based on FH2 domain sequences, the basidiomycote and ascomycote Yeast subtype formins appeared to be related to the holozoan DIAPH/GRID2IP super-group based on a very modestly supported connecting node (bootstrap value 25), while one chytrid formin (SPPG_03524) and one microsporidian formin (Eint_071180) clustered within the holozoan DIAPH/GRID2IP-super-group (Fig 2). Interestingly, when DID-DD sequences were considered, all these formins clustered together as a unified Yeast subtype (Fig 3A), but when the more divergent budding yeast and fission yeast sequences were omitted, the remaining proteins joined a choanoflagellate DIAPH-like formin in a strongly supported group based on DID-DD sequences (bootstrap value 72, Fig 4B). These results suggest that Yeast subtype and fungal DIAPH-like formins may be holomycote representatives of a DIAPH/GRID2IP/Yeast-super-group derived from an ancestral DIAPH-like formin (Fig 5).

Based on FH2 domain and DID-DD sequences, one chytrid formin (SPPG_01270) appeared among the holozoan DAAM proteins (Figs 2–4), but its branch in FH2 domain trees and in a simpler DID-DD tree was also positioned close to well-supported nodes that linked the DAAM, FMNL, and MWHF subtypes into another super-group (Figs 2 and 4). Two alternative explanations for this are that the chytrid formin represents a holomycote DAAM-type protein, or that it is generally related to all three subtypes (DAAM, FMNL, MWHF) due the derivation of the DAAM, FMNL, and MWHF subtypes from a common ancestral formin. Supporting the second possibility, three fonticulid formins also exhibited a mixed relatedness to the DAAM, FMNL, and MWHF subtypes, with the formin H696_01212 appearing DAAM-like based on FH2 domain sequence but FMNL- and MWHF-related based on DID-DD sequence, H696_05026 appearing DAAM-like based on DID-DD sequence but equally related to DAAM, FMNL, and MWHF based on FH2 domain sequence, and H696_04106 lacking an annotated DID-DD sequence but having an FH2 domain also equally related to those three subtypes (Figs 2 and 3). These combined results suggest that an ancestral opisthokont G-DID-DD-FH1-FH2 Drf-type formin gave rise in the holozoan lineage to the DAAM, FMNL, and MWHF proteins, while in the holomycote lineage, the same formin gave rise to a related group of fungal and fonticulid proteins (Fig 5).

Based on FH2 domain sequence, one chytrid formin (SPPG_06650) clustered with the choanoflagellate PTEN-formins with moderate to strong support (bootstrap values 59 and 91 in Figs 2 and 4 A, respectively). The Ensembl annotation for SPPG_06650 predicts this gene encodes an N-terminally truncated FH2 domain. However, the upstream annotated gene (SPPG_06651) is predicted to encode an N-terminal PTEN-like domain followed by an FH1-like proline-rich stretch, and a short sequence homologous to the beginning of an FH2 domain. Considering this, it seems likely that the true full-length chytrid gene encompasses both predicted genes (SPPG_06651_SPPG_06650) to encode a PTP-C2-FH1-FH2 formin similar to the choanoflagellate PTEN-formins (Fig 1). A microsporidian formin (Eint_101200) clustered with PHCF subtype proteins based on its FH2 domain sequence (Fig 2), but its FH2 domain sequence is very divergent, leaving the possibility that this clustering reflects long-branch attraction rather than true relatedness. Supporting this possibility, PHYRE^2^ predicted no PH domains for Eint_101200, but instead predicted an N-terminal PTEN-like domain with PTP- and C2-like folds (Fig 1), suggesting this formin also belongs to the PTEN-formin subtype (indicated by double arrow in Fig 2).

The remaining fungal and fonticulid formins fell into the Fungi-2 subtype in most trees (Figs 2–4). These formins were predicted by PHYRE^2^ to encode a G-DID-DD-FH1-FH2 Drf-type domain organization, with the addition of a Ras-associating (RA) domain at the extreme N-terminus of the fonticulid formin (Fig 1). By FH2 domain, DID, or DID-DD sequence, Fungi-2 subtype formins did not appear to be particularly related to any metazoan subtype.

Summarizing these observations, the common opisthokont ancestor for Holomycota and Holozoa likely encoded at least three formins: a Drf-type G-DID-DD-FH1-FH2 formin that gave rise to the DIAPH/GRID2IP/Yeast super-group formins, a second Drf-type G-DID-DD-FH1-FH2 formin that in holozoans gave rise to the DAAM, FMNL, and MWHF proteins, and a PTP-C2-FH1-FH2 PTEN-formin that was lost from metazoans but retained in some fungi and choanoflagellates (Fig 5). No obvious FHOD, FMN, PHCF, or INF relatives were found among the holomycotes.

### Amoebozoan and apusozoan formins and evidence for a common FHOD/FMN-related ancestral formin

To consider relationships among formins of organisms even more diverged from metazoans, proteins were examined from Apusozoa and Ameobozoa, two sister groups of Opisthokonta within Unikonta, one of the two primary clades of Eukaryota [41]. Twenty-seven formins were examined from the apusozoan *Thecamonas trahens* and the amoebozoan *D*. *discoideum*. As might be expected for organisms so distantly related to metazoans, many of their FH2 domains did not cluster with metazoan subtypes in phylogenetic trees. The striking exceptions to this were several formins from both organisms that were related to the FMN and FHOD subtypes. Branches leading to these apusozoan and amoebozoan formins were positioned close to nodes that joined the metazoan FMN and FHOD subtypes in a strongly supported super-group (bootstrap values 59 and 72 in Figs 2 and 4A, respectively).

The N-terminal domain organizations of FHOD and FMN proteins differ dramatically (Fig 1). In this regard, amoebozoan and apusozoan FHOD/FMN-related formins appeared more similar to the FHOD subtype proteins. PHYRE^2^-based analysis of four amoebozoan and apusozoan FHOD/FMN-related formins predicted N-terminal G2 and DID structures, as well as a DD, to yield a basic domain organization G2-DID-DD-FH1-FH2 shared by these proteins (Fig 1). Moreover, the DIDs of these formins clustered with the FHOD subtype proteins in a very strongly supported group (bootstrap value 99) (Fig 3B). Supporting these computational predictions, an NMR-based structure determination of the N-terminus of one of these formins (FORC of *D*. *discoideum*) had previously shown that it adopts an FHOD-like GTPase-binding (G2) fold [44].

One divergent feature of two apusozoan FHOD/FMN-related proteins was a predicted PH domain at their extreme N-terminus (Fig 1), but this was not observed in any amoebozoan or holozoan homolog. Among opisthokont formins, PH domains are only found in PHCFs. The absence of strong similarity between the FH2 sequences of PHCF and FHOD/FMN proteins, and the absence of PH domains from amoebozoan and holozoan homologs, argue against these PH domains resulting from a shared ancestry with PHCFs, but are likely an apusozoan innovation.

These results suggest that the common unikont ancestor for Opisthokonta, Apusozoa, and Amoebozoa encoded an ancestral FHOD/FMN-related formin with a G2-DID-DD-FH1-FH2 domain organization (Fig 5). Among apusozoans, an N-terminal PH domain was acquired. Among opisthokonts, this formin appears to have been lost from holomycotes, but was duplicated in holozoans. One of these holozoan duplicates lost its DD to become the FHOD subtype with G2-DID-FH1-FH2 domain organization, while for the other duplicate, the entire N-terminus was replaced with novel sequence to produce the X-FH1-FH2 FMN subtype (Fig 5).

Most remaining apusozoan and amoebozoan formins featured a Drf-type G-DID-DD-FH1-FH2 domain organization, often supplemented with additional domains and motifs unique to particular isoforms [9,11]. None of these formins grouped strongly with a metazoan subtype based on FH2 domain or DID-DD sequences (Figs 2–4). It is possible that the long evolutionary separation of these organisms from opisthokonts erased evidence of shared origins for distinct Drf subtypes. Alternatively their common unikont ancestor may have encoded a single Drf-type formin that multiplied independently in each lineage (Fig 5).

### Bikont formins and evidence for a PTEN-formin in the common ancestor of unikonts and bikonts

The organisms considered so far belong to the major eukaryotic clade Unikonta. A previous study examined formins from species of the second major clade, Bikonta, but observed no particular relatedness between those formins and any unikont subtype [12]. However, many bikont formins, particularly from species belonging to Plantae and Heterokonta, have a domain organization PTP-C2-FH1-FH2, similar to the fungal and choanoflagellate PTEN-formins identified here [12,45]. To reexamine the relationships between unikont and bikont proteins, forty bikont formins were identified from two representatives of Plantae, *Arabidopsis thaliana* and *Physcomitrella patens*, and two representatives of Heterokonta, *Phaedactylum tricornutum* and *Phytophthora ramorum* (S1 Table). Their FH2 domain sequences were aligned with those from the unikonts, and a ML tree was estimated and tested by bootstrap analysis (S1 Fig). In many respects, this tree reproduced what had been found previously [12]. Plant formins gathered into three previously described subtypes, Class I, Class II, and Class III, with modest kinship between Class I and Class III subtypes (bootstrap value 48). Most heterokont formins fell into a single weakly supported group that was associated with Class II plant formins through a poorly supported node (bootstrap value 15). Also supporting what was previously observed (Grunt et al., 2008), there was no apparent relationship between any bikont formin subtype and any particular unikont formin subtype based on estimated FH2 domain phylogeny (S1 Fig).

**Fig 6:**
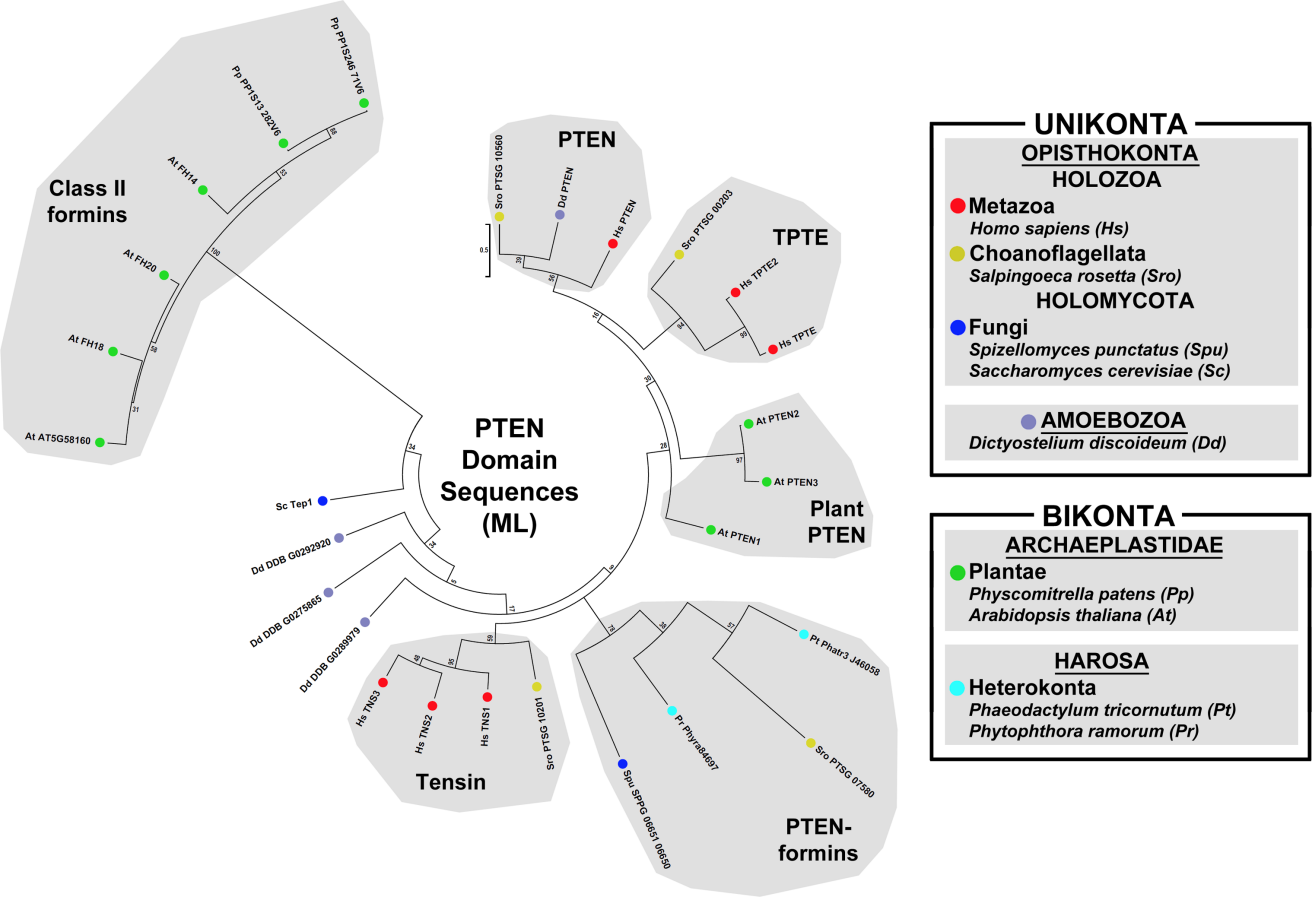
Unrooted ML phylogenetic tree of PTEN-like domains from unikont and bikont proteins. Evolutionary histories for 27 PTEN-like domain sequences of formin and non-formin proteins from the indicated species were inferred by the ML method using the LG + G model for 80 positions that were fully occupied in all sequences. Major groups of related PTEN-like domains identified include unikont PTEN proteins, holozoan transmembrane phosphatase with tensin homology (TPTE) proteins, holozoan tensin proteins, and two groups of PTEN-formins, one that includes plant Class II formins, and another that includes opisthokont and heterokont PTEN-formins. All bootstrap values are indicated, and the scale bar indicates the number of substitutions per site for branch lengths.

To probe for potential relatedness specifically between the unikont and bikont PTEN-formins, PTEN-like domain sequences of ten formins and seventeen non-formin proteins were aligned and a ML phylogenetic tree was estimated (Fig 6). Again, this tree reproduced several of results of a similar PTEN-like phylogenetic analysis of bikont and unikont proteins [12], showing no particular relationship between plant and heterokont PTEN-formins, or between PTEN-formins and non-formins. However, a novel result was a clustering of the fungal and choanoflagellate PTEN-formins with the heterokont PTEN-formins as a single subtype behind a strongly supported node (bootstrap value 78) (Fig 6). This suggests unikont PTEN-formins and at least some of the bikont PTEN-formins share a common ancestry (Fig 5).

### Model for the origins of metazoan formin diversity

From this analysis and building on the previous work of others, one possible model is presented here for the pattern of formin diversification that resulted in the extant metazoan formin subtypes (Fig 5). Based on the presence of Drf-type formins in unikonts and bikonts, a previously proposed model for the diversification of the plant formins had suggested the last common eukaryotic ancestor for the unikonts and bikonts encoded at least one Drf with G-DID-DD-FH1-FH2 domain organization [12]. Based on the additional shared presence of related PTEN-formins among unikonts and bikonts (Figs 1 and 6), the model presented here begins with at least two formins in the last common eukaryotic ancestor, including a Drf and a PTEN-formin.

Among the unikonts, the Drfs are found in apusozoans, amoebozoans, and opisthokonts, suggesting the ancestral Drf was inherited by each of these lineages. Additionally, these groups of unikonts also generally share a group of non-Drf proteins that resemble Drfs, but are characterized by a substitution of the Drf-type G domain for a structurally dissimilar G2 domain. Based on the similar domain organizations of Drfs (G-DID-DD-FH1-FH2) and these non-Drfs (G2-DID-DD-FH1-FH2), arose from a duplication and divergence of an ancestral Drf. The presence of such non-Drfs among apusozoans, amoebozoans, and opisthokonts suggests this duplication and divergence occurred early in the unikont lineage, before the divergence of those three groups. Based on an absence of apparent further similarity of subgroups of the Drfs and non-Drfs of these organisms, this model suggests the ancestral Drfs and non-Drfs further multiplied and diverged independently within the apusozoans, amoebozoans, and opisthokonts.

Within the opisthokonts, the ancestral Drf likely diversified early, explaining the observation that holomycotes (including the fungi) and holozoans (including metazoans and choanoflagellates) share several related groups of Drfs. One ancestral opisthokont Drf (Drf1) was likely similar in sequence to the metazoan DIAPH formins. Thus, DIAPH-like formins can be found broadly among the fungi, including the well-studied formins of the budding and fission yeasts. Within the metazoan lineage, the ancestral DIAPH-like formin appears to have duplicated very early, just after the divergence of metazoans from choanoflagellates, to give rise to the conserved DIAPH subtype and to the GRID2IP subtype which acquired a novel PDZ/HN domain-rich N-terminus.

A second ancestral opisthokont Drf-type protein (Drf2) appears to have diversified independently in the holozoans and holomycotes, to give rise to DAAM, FMNL, and MWHF subtypes in metazoans and choanoflagellates, and to a group of holomycote formins that show intermediate relatedness to the DAAM/FMNL/MWHF subtype proteins. Additional ancestral opisthokont Drfs were also likely present to give rise to the formins of the holomycote Fungi-2 subtype, and possibly the INF subtype formins of metazoans and choanoflagellates. The model in Fig 5 includes a speculative suggestion that INF and Fungi-2 formins share a common origin from a putative third opisthokont Drf (Drf3), but absence of strong similarity between these two formin groups makes it at least as likely that they derive from distinct ancestral proteins.

Among the opisthokonts, the G2-DID-DD-FH1-FH2 non-Drf appears to have been lost from the holomycotes, but duplicated and diverged in the holozoan lineage. One duplicate lost its dimerization motif to become the FHOD subtype proteins with the domain organization G2-DID-FH1-FH2. The other duplicate lost all of its N-terminal domains and adopted a novel N-terminus, producing the FMN proteins with domain organization X-FH1-FH2. Thus, based on domain organization, the FMN proteins are unrecognizable as descendants of the ancestral non-Drf. However, the clear relatedness of the FMN FH2 domain sequences to those of FHOD proteins and ameobozoan and apusozoan non-Drfs strongly supports this origin. The presence of distinct FHOD and/or FMN subtype proteins in all the holozoans examined here suggests that the duplication and divergence of the ancestral non-Drf occurred in the holozoan lineage before the divergence of the metazoans, choanoflagellates and filastereans.

Thus, this model suggests that eight of the nine metazoan formin subtypes ultimately arose from a G-DID-DD-FH1-FH2 Drf and a G2-DID-DD-FH1-FH2 non-Drf that were present in the last common unikont ancestor of the apusozoans, amoebozoans, and opisthokonts. The model suggests the PTEN-formin was also present in this ancestral unikont. Based on the single representatives of Apusozoa and Amoebozoa examined here, the PTEN-formin might have been lost from the lineages leading to those groups. Among the opisthokonts, PTEN-formins appear to have been lost among several lineages, including the metazoans, but were retained among others, including the fungi and choanoflagellates.

The origin of the ninth metaozoan subtype, the PHCFs, is unclear. PHCF homologs were found in representatives from all the holozoan groups examined here, suggesting that the last common holozoan ancestor encoded a PHCF. However, among the formins sampled here, the PH-PH-FH1-FH2-PH domain structure is unique to PHCFs, and the FH2 domain sequences of PHCFs show no particularly strong relatedness to other eukaryotic formin subtypes. One possible explanation for this is that PHCFs might have arisen through horizontal transfer into the holozoan ancestor from some other lineage. The presence of formins with PH domains among heterokonts (Grunt et al., 2008) points to a possible source. Alternatively, the ancestral PHCF might have arisen through a novel rearrangement that combined an FH2 domain with a series of three PH domains. This, coupled to a particularly high degree of sequence divergence for this FH2 domain might have obscured its relationship to other formins. Perhaps with identification of additional non-metazoan formins, the relationship between the PHCFs and the remaining members of the formin family tree will be discovered.

## Conclusions

While previous phylogenetic studies have delineated conserved subgroups of formins within particular kingdoms of organisms, they have generally failed at revealing relationships between formins from different kingdoms. With the availability of genomes from organisms more broadly distributed across the eukaryotic family tree, it is now becoming possible to trace some of these relationships. As shown here, the origins of the formin subtypes of the metazoans are deep. The evidence suggests a gradual process of formin duplication and divergence occurred over time during the evolution of metazoans from an ancient unikont ancestor.

Formin diversity seems likely to contribute to the ability of metazoan cells to assemble a wide variety of cytoskeletal architectures, particularly actin-based ones. Formin diversity may also have contributed to the evolution and diversification of actin, itself, in metazoans. Actin plays essential roles in the cell, and interacts with bewildering number of proteins, placing strong constraints on its ability to evolve. It has been hypothesized that the ability of cells to assemble distinct populations of actin filaments, which provide alternative selective environments for actin, may have been a precondition to allow for the evolution of multiple actin isoforms in metazoans [46]. Considering the very early roots of formin diversity shown here, it seems likely formin evolution may have been an early contributor to setting the stage for the evolution and diversification of actin and other cytoskeletal proteins in metazoans.

## Supporting information

S1 Table List of formins used in this study. (XLSX)

S1 Text Multiple sequence alignments in interleaved format. (TXT)

S1 Fig Unrooted ML phylogenetic tree of FH2 domains from unikont and bikont proteins. Evolutionary histories for 180 FH2 domain sequences from the indicated species were inferred by the ML method using the LG + G model for 270 positions that were occupied ≥ 95% of FH2 sequences. Most previously identified major groups of unikont formins were reproduced here, as were the three plant formin subtypes (Class I, Class II, Class III), and a group of heterokont formins that was associated with the Class II plant formins. Formins that encode N-terminal PTEN-like domains are indicated with red arrows. All bootstrap values are indicated, and the scale bar indicates the number of substitutions per site for branch lengths.
(TIF)
