# Supplementary figures and images for: Probing the origins of metazoan formin diversity: Evidence for evolutionary relationships between metazoan and non-metazoan formin subtypes
Source: PLoS One. 2017 Oct 5;12(10):e0186081. doi: 10.1371/journal.pone.0186081 (PMC5628938; doi:10.1371/journal.pone.0186081)

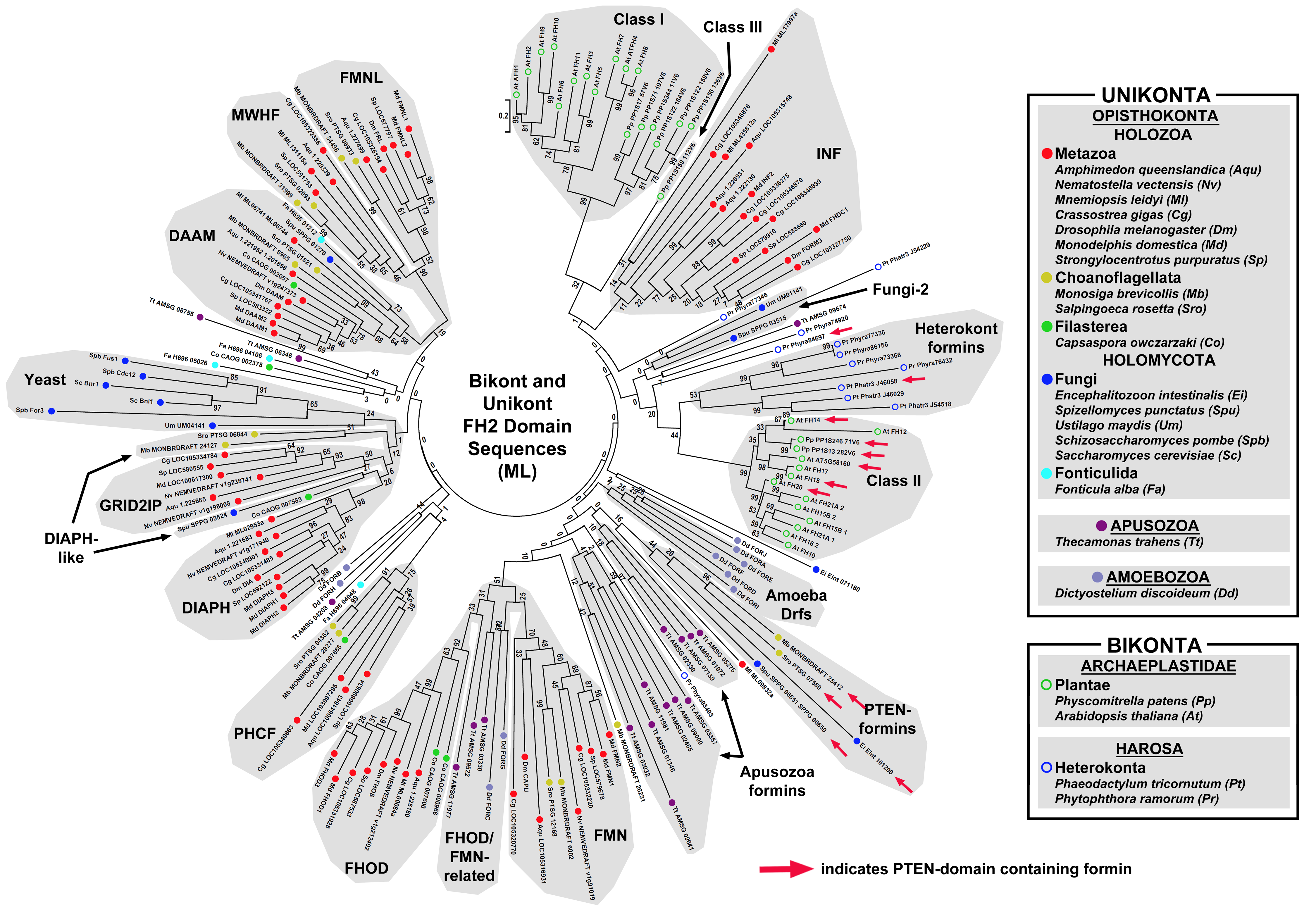

Supplement: S1 Fig — Evolutionary histories for 180 FH2 domain sequences from the indicated species were inferred by the ML method using the LG + G model for 270 positions that were occupied ≥ 95% of FH2 sequences. Most previously identified major groups of unikont formins were reproduced here, as were the three plant formin subtypes (Class I, Class II, Class III), and a group of heterokont formins that was associated with the Class II plant formins. Formins that encode N-terminal PTEN-like domains are indicated with red arrows. All bootstrap values are indicated, and the scale bar indicates the number of substitutions per site for branch lengths. (TIF) [file pone.0186081.s003.tif]
